# A Grey BP Neural Network-Based Model for Prediction of Court Decision Service Rate

**DOI:** 10.1155/2022/7364375

**Published:** 2022-04-14

**Authors:** Gang Zhao, Huibin Shi, Jifa Wang

**Affiliations:** School of Management, Shenyang University of Technology, No. 111, Shenliao West Road, Economic & Technological Development Zone, Shenyang 110870, China

## Abstract

The judgment service rate is an important index to reflect the fairness of the judgment of legal cases in a certain area, which is of great significance to verify the accuracy of a court judgment. In this paper, a grey neural network model combining grey system theory and BP neural network algorithm is proposed to predict the index. Analyze the judgment service rate of the court judgment system, and build a prediction system based on the completion rate, completion rate, plaintiff satisfaction, defendant satisfaction, litigation time, property preservation cycle, document delivery time, implementation information disclosure rate, and other key indicators. Through example analysis, it is proved that the combined model of the grey prediction model and BP neural network has a small error and good simulation effect on the prediction of court decision-making service rate, which can better promote the development of court and society.

## 1. Introduction

In judicial statistics, the settlement methods of first instance cases include mediation and judgment. According to the law, in principle, there is no appeal in the case settled through mediation. It can be seen that the appeal case arises from the case settled by the judge. The percentage of appeal cases in the cases closed in the first instance is the appeal rate, and on the contrary, it is the rate of serving judgment and interest. To some extent, the rate of serving judgment and interest litigation reflects the case handling ability of case handling judges, especially the ability to work as litigants. In building a harmonious society, it is very important to continuously improve the rate of serving judgment and interest litigation of first instance cases. It reflects the recognition, satisfaction, and acceptance of the litigants to the court judgment from one side [1].

The rate of judgment service is an important basic indicator of the trial quality of a court, and it is also an important embodiment of the judicial ability and mass work ability of the court. To a certain extent, it comprehensively and objectively reflects the overall quality status of the court's case trial work, which is conducive to the court to adjust its working ideas in time, strengthen the quality consciousness, high-quality consciousness, responsibility consciousness of the judges, solidly control the case quality, and improve the judicial ability. Courts all over the world attach great importance to this index and believe that it can show the importance of trial quality management, judges' case handling ability, and the error-free rate of judgment. The level of judicial ability directly affects the fairness and impartiality of the judgment and the rate of interest litigation of the parties. A judge with profound legal foundation and strong professional ability makes a fair ruling in strict accordance with the legal procedures in the judgment which has high recognition and acceptance of the judgment and strong willingness to serve the judgment, so the possibility of appeal is greatly reduced. Therefore, the current literature is more concerned about the prediction of the judgment service rate. The main method models are grey GM (1, 1) model [2, 3], grey Markov chain model [4, 5], multiple linear regression model [6, 7], etc., however, these method models do not have the ability of self-learning and self-adaptation, resulting in a large error of calculation results. The artificial neural network does not need to determine the mathematical equation of the mapping relationship between the input and output in advance. It only learns some rules by its own training and gets the result closest to the expected output value when the input value is given. As an intelligent information processing system, the core of the artificial neural network is the algorithm. The BP neural network is a multilayer feedforward network trained according to error back propagation. Its algorithm is called the BP algorithm. Its basic idea is the gradient descent method, which uses gradient search technology to minimize the error mean square deviation of the actual output value and expected output value of the network. This paper uses the grey neural network algorithm to predict the judgment service rate of the court. The grey neural network combines the grey system theory and the BP neural network algorithm, has the ability of self-learning and self-adaptation, can constantly modify the error, and scientifically predicts the service judgment obedience rate, which will help to improve the speed and accuracy of judicial justice.

## 2. Literature Review

The development of science and technology is gradually promoting the arrival of the era of artificial intelligence. The competition between regions is not just economic competition. While the economy is developing rapidly, people also pay more attention to social fairness and justice. A fair and just social environment is an indispensable condition for the long-term, stable, and healthy development of society. In today's society, big data and artificial intelligence have provided a very important platform [8] for enterprise development, talent training, and social governance, and they have gradually penetrated into various social fields. Especially in the judicial field, [9, 10] big data has become more and more widely applied, playing a positive role and great contribution to maintaining social order and stability.

With the continuous development of the economy and society, intelligent judicial systems at home and abroad are also constantly improving and developing. Through the use of advanced management theory and scientific and technological means, it can provide a favorable guarantee for judicial trial [11]. In various fields of society, Luo et al. [12] analyzed the reasons why the grey model GM (1, 1) often produces errors in the simulation data and found that the background value structure method of the grey model GM (1, 1) has an important impact on the prediction and adaptability of the model. The optimized background value makes the GM (1, 1) model have a better fitting and prediction accuracy. Li et al. [13] used the grey system model and its principle to predict the settlement of soft soil foundation of class I highway. By calculating the model accuracy evaluation index, it shows that this method has a small fitting error and high prediction accuracy. Zhang and Jiao used the grey GM (1, 1) model and the BP neural network to predict the demand for the number of employees of an enterprise (ZTE) [14] and predicted the overall demand for the number of employees of ZTE in the next three years by combining the results obtained by two different prediction methods. Yin and Chen [15] used the BP neural network model to predict the number of babies born in a certain area. The prediction results are very close to the actual situation, which provides an important basis for the local government to formulate relevant policies. Xu and Gu [16] used the BP neural network to predict the product cost and expounded the benefits of using this method to predict the product cost. Combined with practical cases, it was proved that the prediction results were very close to the actual value. Liu and Jiang [17] used the grey GM (1, 1) model and the BP neural network model to predict the future development trend of China's population, and the prediction results have high prediction accuracy. Based on the grey correlation analysis and improved method, Jiang and Wan [18] selected the data of China's cargo transportation volume and its influencing factors from 1999 to 2013 to predict and test China's cargo transportation volume from 2014 to 2018. The error of the prediction results is very small, which gives us great inspiration. The prediction based on the grey GM (1, 1) model and the BP neural network model has been applied to many aspects of economic and social life. The accuracy of the two prediction models is constantly improving, and different methods should be adopted according to different situations.

In the judicial field, the rate of serving judgment and interest litigation has become one of the important standards of whether the court judgment is fair. The demand prediction of the rate of serving judgment can provide a reference for the court trial. With the deepening of research, the problems of the grey GM (1, 1) model prediction and BP neural network prediction have been continuously improved, and the prediction results are more scientific and reasonable, which is suitable for the prediction of judgment service rate. In this paper, by comparing the two prediction methods to improve the scientificity and rationality of prediction, it has strong practical significance.

## 3. Model Establishment

### 3.1. Theoretical Basis

The grey system model has the characteristics of insufficient sample data, uncertain information, no self-learning, self-adaptation ability, and a weak ability to solve nonlinear problems. The slight change of sample data will make the whole grey system model continuously supplement new information and remove the old information with little meaning to make the model change dynamically. The farther the prediction time is, the worse the prediction accuracy is. The BP neural network model imitates the working mode of biological neurons. It has the characteristics of sufficient sample data, strong self-learning ability, self-adaptation ability, and a strong ability to solve nonlinear problems. The combination of the grey system model and the BP neural network model can make up for each other's shortcomings and enhance the ability to solve problems. Combining the grey system model with the BP neural network model and using their respective characteristics, a grey neural network prediction model with stronger stability, higher prediction accuracy, and faster problem processing speed is established.

In this paper, the data is processed first, and then the grey system model establishes samples for the processed data. Finally, the sample data is predicted using the self-learning and self-adaptability of the BP neural network. The process is as follows: firstly, normalize and accumulate the data, respectively, then the grey system generates the processed data into sample data, inputs the sample data into the BP neural network for training, and finally outputs the prediction results of the trained BP neural network.

### 3.2. Grey Neural Network Model

(1) The GM (1, 1) 1n model is established according to the relevant theory of grey system.
A multidimensional grey system model with *N* input variables and 1 output variable is established.
(2) Normalize the data.
In this paper, the deviation standardization method is used to transform the original sequence.
  (1) $$\begin{array}{c} a_{j}=\frac{b_{i}-\min\left\{b_{1},b_{2},\ldots ,b\right\}}{\max\left\{b_{1},b_{2},\ldots ,b_{n}\right\}-\min\left\{b_{1},b_{2},\ldots ,b_{n}\right\}}. \end{array}$$
(3) Accumulate the normalized data.
Let the normalized original sequence be *A*^(0)^={*a*_1_^(0)^, *a*_2_^(0)^,…, *a*_*n*_^(0)^}.
  (2) $$\begin{array}{c} a_{k}^{\left(1\right)}=\sum_{i=1}^{k}a_{i}^{\left(0\right)}\left(k=1,2,\cdots ,n\right). \end{array}$$
where
(4) Establish grey differential equation.
The differential equation of establishing a grey neural network model is as follows:
  (3) $$\begin{array}{c} \frac{da_{t}^{\left(1\right)}}{dt}+ca_{1}^{\left(1\right)}=d_{1}a_{2}^{\left(1\right)}+d_{2}a_{3}^{\left(1\right)}+\cdots d_{n-1}a_{n}^{\left(1\right)}. \end{array}$$
where c and d1, d2,…, dn are the coefficients of the differential equation.
The time response formula of (4) is as follows:
  (4) $$\begin{array}{c} x\left(t\right)=\left(a_{1}^{\left(1\right)}\left(0\right)-\frac{1}{c}\sum_{i=2}^{n}d_{i=1}a_{i}^{\left(1\right)}\left(t\right)\right)e^{-ct}+\frac{1}{c}\sum_{i=2}^{n}d_{i=1}a_{i}^{\left(1\right)}\left(t\right). \end{array}$$
  (5) $$\begin{array}{c} f=\frac{1}{c}\sum_{i=2}^{n}d_{i=1}a_{i}^{\left(1\right)}\left(t\right). \end{array}$$
Then, formula (4) can be transformed into the following:
  (6) $$\begin{array}{c} x\left(t\right)=\left(a_{1}^{\left(1\right)}\left(0\right)-f\right)-a_{1}^{\left(1\right)}\left(0\right)\frac{1}{1+e^{-ct}}+2f\frac{1}{1+e^{-ct}}\left(1+e^{-ct}\right). \end{array}$$
(5) The grey neural network is obtained by mapping the time response formula to the BP neural network

**Figure 1:**
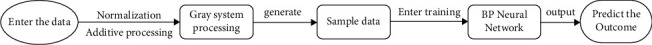
Algorithm flow chart.

**Figure 2:**
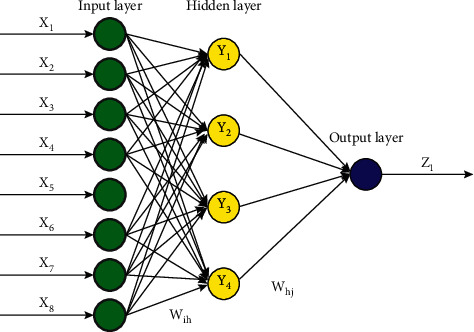
BP neural network.

Upon mapping equation (6) to the extended BP neural network shown in Figure 1, the grey neural network with 8 input parameters and 1 output parameter will be obtained by establishing the GM (1, 1) 18 model. The extended BP neural network has 8 input nodes. Through experiments, it is found that the prediction result is the most accurate when the number of hidden layer neurons is 4. The stimulus function of the hidden layer is the S-type tangent function, which is a sigmoid function. The sigmoid function has the properties of monotonic increase and inverse function monotonic increase, and its value is any number among 0, 1, and 11. The stimulus function of the output layer is a purelin linear function, which is a linear transfer function with the characteristic that the input is equal to the output. The training function is the train function, which is the training function of the neural network. Its function is to realize the self-learning and self-adaptation of neural network and constantly update the neural network parameters until the minimum error or the maximum learning steps are reached, as shown in Figure 2 [19].

### 3.3. Grey Neural Network Algorithm Steps

(1) Use the training data to initialize the parameters of the grey neural network.
  (7) $$\begin{array}{c} w_{11}=1,w_{21}=-a_{1}^{\left(1\right)}\left(0\right),w_{22}=\frac{2d_{1}}{c},w_{23}=\frac{2d_{2}}{c},\ldots ,w_{2n}=\frac{2d_{n-1}}{c}. \end{array}$$
  (8) $$\begin{array}{c} w_{11}=w_{21}=\cdots =w_{3n}=1+e^{-ct}. \end{array}$$
Set the threshold of the LD layer according to the definition of the threshold.
  (9) $$\begin{array}{c} \theta =\left(1+e^{-ct}\right)\left(f-a_{1}^{\left(1\right)}\left(0\right)\right). \end{array}$$
(2) For each training data, calculate the output of each layer.
The output of the LA layer is as follows:
  (10) $$\begin{array}{c} c=w_{11}t. \end{array}$$
The output of the LB layer is as follows:
  (11) $$\begin{array}{c} d=\frac{1}{1+e^{-w_{11}t}}. \end{array}$$
The output of the LC layer is as follows:
  (12) $$\begin{array}{c} g_{1}=d_{11},g_{2}=a_{2}^{\left(1\right)}dw_{22},g_{3}=a_{3}^{\left(1\right)}dw_{23},\cdots ,g_{n}=a_{n}^{1}dw_{2n}. \end{array}$$
The output of the LD layer is as follows:
  (13) $$\begin{array}{c} h={\mathrm{dw}}_{31}g_{1}+{\mathrm{dw}}_{32}g_{2}+\cdots +{\mathrm{dw}}_{3n}g_{n}-\theta_{a}^{\left(1\right)}\left(t\right). \end{array}$$
(3) Calculate the error between the predicted value and the actual value of the grey neural network.
The error of the LD layer is as follows:
  (14) $$\begin{array}{c} \delta =h-a_{1}^{\left(1\right)}\left(t\right). \end{array}$$
The error of the LC layer is as follows:
  (15) $$\begin{array}{c} \delta_{1}=\delta \left(1+e^{-w_{11}t}\right),\delta_{2}=\delta \left(1+e^{-w_{11}t}\right),\cdots ,\delta_{n}=\delta \left(1+e^{-w_{11}t}\right). \end{array}$$
The error of the LB layer is as follows:
  (16) $$\begin{array}{c} \delta_{n=1}=d\left(1=d\right)\left(w_{21}\delta_{1}+w_{22}\delta_{2}+\cdots +w_{2n}\delta_{n}\right). \end{array}$$
(4) Adjust the weight of the grey neural network according to the prediction error.
The adjusted weight from the LB layer to the LC layer is as follows:
  (17) $$\begin{array}{c} w_{21new}=-a_{1}^{\left(1\right)}\left(0\right),w_{22new}=w_{22}-u_{1}\delta_{2}d,\cdots ,w_{22new}=w_{2n}-u_{n-1}\delta_{n}d, \end{array}$$
The adjusted weight from the LA layer to the LB layer is as follows:
  (18) $$\begin{array}{c} w_{11\text{new}}=w_{11}+ct\delta_{n+1}. \end{array}$$
(5) Adjust the threshold of the grey neural network according to the prediction error.
The adjusted threshold is as follows:
  (19) $$\begin{array}{c} \theta =\left(1+e^{-w_{11}newt}\right)\left(\frac{w_{22new}}{2}\;a_{2}^{\left(1\right)}\left(t\right)+\frac{w_{22new}}{2}\;a_{3}^{\left(1\right)}\left(t\right)+\cdots +\frac{w_{22new}}{2}\;a_{n}^{\left(1\right)}\left(t\right)-a_{1}^{\left(1\right)}\left(0\right)\right). \end{array}$$
(6) Judge whether the training of the grey neural network reaches the goal of the expected error. If not, return to step (2) for training again.

## 4. ABC Court Judgment Acceptance Rate Grey Neural Network Prediction Model

### 4.1. Factors Influencing the Rate of the Service of Court Judgments of the ABC Court

Forecasting the rate of the acceptance of judgments requires the consideration of appropriate factors. In general, no case predicts the rate of judgment obedience based on only one factor, however, a comprehensive forecast is based on multiple related factors. Studies [19–22] have a higher impact on the judgment acceptance rate of the following eight factors: the case closure rate, the completion rate, the plaintiff satisfaction rate, the defendant satisfaction rate, the litigation time, the property preservation cycle, the time of service of documents, and the enforcement information disclosure rate.

 
*Case Closure Rate*. Case closure refers to the special activities of the investigating organs in the course of handling criminal cases to achieve that the facts and circumstances of the crime are clear, the evidence is credible and sufficient, the nature and crime of the crime are determined to be accurate, the legal procedures are complete, and the investigation organs make conclusions about the case and put forward handling opinions or make handling arrangements. The case closure rate refers to the ratio of the number of cases closed to the number of cases filed.
 
*Completion Rate*. Enforcement generally refers to the activities carried out in accordance with law by the court to realize the content determined by the judgment, ruling, mediation document, etc., which has already taken effect, as well as the modification of enforcement in the process of enforcement. The enforcement rate refers to the ratio of the cases that have already been enforced in the people's courts' enforcement cases to all enforcement cases, reflecting the final results of the court's enforcement work.
 
*Litigation Time.* The limitation of actions is a legal fact that can cause changes in civil legal relations, also known as the extinction of the limitation period, which refers to the limitation system in which the right holder does not exercise the rights within a certain period of time, i.e., the holder loses the benefits of the request to a certain extent. The main purpose of establishing the statute of limitations system is to promote the stability of legal relations, promptly end the uncertain state of rights and obligations, stabilize the legal order, and reduce transaction costs.
 
*Plaintiff Satisfaction Rate*. It refers to a person who, in order to protect his legitimate rights and interests, files a lawsuit with the court in his own name, thus causing the litigation procedure to occur. The plaintiff's satisfaction rate refers to the ratio of the number of cases satisfactory to the total number of cases.
 
*Satisfaction rate of the defendant*. The satisfaction rate of the defendant refers to the ratio of the number of cases satisfied by the plaintiff to the total number of cases.
 
*Property preservation Cycle*. The effect of property preservation is generally maintained until the date on which the judgment becomes effective and the application for enforcement is made to the court. The period of property preservation depends on the type of property, and if the preservation bank seals it, the period of preservation shall not exceed one year. If a movable property is preserved, the period of preservation shall not exceed 2 years. If the real property is preserved, the period of preservation shall not exceed 3 years. If no renewal or freezing measures are taken at the expiration date, the preservation measures will automatically become invalid.
 
*Time of Delivery of Paperwork*. Service by public announcement means that the court makes the content of the litigation document public by means of an announcement, and after a statutory period, it is deemed to be served. The service of notice is a kind of presumed service, and after the court issues an announcement, it should keep the litigation documents that should be served so that the person who serves it can receive them at any time. Upon the expiry of the notice period, it shall be deemed to have been served.
 
*Implementation of Information Disclosure Rates*. The disclosure of enforcement refers to the people's court making public the enforcement process and enforcement procedures of the case. The enforcement information disclosure rate refers to the ratio of the enforcement information disclosure data to the number of enforcement cases.

**Figure 3:**
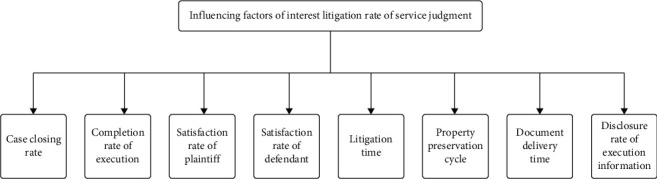
Factors influencing the rate of judgment service.

All eight factors in Figure 3 [23] are essential in predicting sentence compliance rates. They can affect the rate of judgment and they do not interact. At present, there is little research on the predicted grey model systems in the judicial field, and the existing grey system models can be improved. Therefore, the grey system model can be combined with the BP neural network model to compensate for the deficiency and predict satisfaction based on these 8 factors.

### 4.2. ABC Court Judgment Acceptance Rate Influencing Factors Data Preparation

Define the dependent variable (judgment acceptance rate) as *y*, the 8 independent variables as (the total number of adjudicated cases/cases) X1, (enforced/adjudicated cases) X2, (plaintiff satisfaction cases/total cases) X3, (defendant satisfaction cases/total number of cases) X4, (litigation time) X5, (property preservation period) X6, (time of service of documents) X7, and (enforcement information disclosure data/enforcement cases) X8. ABC court statistics for 2008 to 2020 are shown in Table 1.

### 4.3. Metric Value Processing

#### 4.3.1. Commonly Used Data Processing Methods

(1) The standardized treatment is as follows:
  (20) $$\begin{array}{c} x_{ij}^{*}=\frac{x_{ij}-{\bar{x}}_{j}}{s_{j}}, \end{array}$$
where (*J* = 1, 2,…, M) are the (sample) average value and (sample) standard deviation of the j^th^ index observation value, respectively, which is called the standard observation value.
(2) The extreme value processing method is as follows:
  (21) $$\begin{array}{c} \overset{*}{\underset{ij}{x}}=\frac{x_{ij}-m_{j}}{M_{j}-m_{j}}. \end{array}$$
Among them, *M*_*j*_=max{*x*_*ij*_}, *m*_*j*_=min{*x*_*ij*_} (the following formulas are the same).
(3) The linear proportional method is as follows:
*x*_*ij*_^*∗*^=*x*_*ij*_/*x*_*j*_′*X*_*j*_′ is a definite special point.
Generally, it can be taken as $m_{j}M_{j}\mathrm{or}\bar{X_{j}}$.
(4) The normalization method is as follows:
  (22) $$\begin{array}{c} \overset{*}{\underset{ij}{x}}=\frac{x_{ij}}{\sum_{I=1}^{N}x_{ij}}. \end{array}$$
(5) The vector norm method is as follows:
  (23) $$\begin{array}{c} \overset{*}{\underset{ij}{x}}=\frac{x_{ij}}{\sqrt{\sum_{i=1}^{n}x_{ij}^{2}}}. \end{array}$$
(6) The efficacy coefficient method is as follows:
  (24) $$\begin{array}{c} \overset{*}{\underset{ij}{x}}=c+\frac{x_{ij}-m_{j}^{\prime }}{m_{j}^{\prime }-m_{j}^{\prime }}\times d. \end{array}$$

In the formula, *M*_*j*_′*M*_*j*_′ are the satisfactory and impermissible values of index *x*_*j*_, respectively. C and D are known normal numbers. C is used to “translate” the transformed value, and C is used to “enlarge” or “reduce” the transformed value.

#### 4.3.2. Triangular Fuzzy Function

*(1) Theoretical Basis of Fuzzy Sets*. Zadeh proposed the fuzzy set theory (FST) to solve the problem of fuzzy phenomena in practice. Let U be the universe and $\tilde{A}$ be a subset of U. For any element x ∈ U and function µ $\tilde{A}$: U ⟶ [0, 1], a value µ $\tilde{A}$ (*x*) ∈ U is specified corresponding to it. The value of µ $\tilde{A}$ (*x*) at element *x* reflects the degree to which element *x* belongs to $\tilde{A}$. Set $\tilde{A}$ is called fuzzy subset, and µ $\tilde{A}$ (*x*) is called the membership function of $\tilde{A}$. The greater the value of µ $\tilde{A}$ (*x*), the higher the degree to which element *x* belongs to $\tilde{A}$.

A triangular fuzzy number (TFN) is defined as a triple $\tilde{A}$ = (a, B, *c*), where a < b < *c*, and all are real numbers. Its membership function is as follows:

(25) $$\begin{array}{c} \left\{\begin{array}{cc} \frac{x-a}{b-a}, & a\leq x\leq b\\ \frac{c-x}{c-b}, & b\leq x\leq c\\ 0, & \text{else} \end{array}\right). \end{array}$$

**Figure 4:**
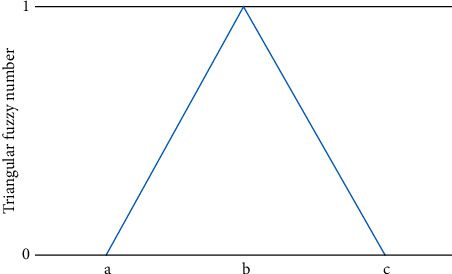
Triangular fuzzy number.

The membership function diagram is shown in Figure 4 [24].

In this paper, the court can express fuzzy opinions in the form of triangular fuzzy numbers. The reason for using triangular fuzzy numbers is that the representation method is intuitive and easy to use.

*(2) Triangular Fuzzy Number Fuzzy Operation*. Two triangular fuzzy numbers $\tilde{A}=\left(a,b,c\right)\mathrm{and}=\left(e,f,g\right)$ are known, and the four basic operations are as follows:

(1) The sum of two fuzzy numbers ⊕ is as follows:
  (26) $$\begin{array}{c} \tilde{A}⊕\tilde{B}=\left(a+e,b+f,c+g\right),a\geq 0,e\geq 0. \end{array}$$
(2) The product of two fuzzy numbers ⊗ is as follows:
  (27) $$\begin{array}{c} \tilde{A}⊗\tilde{B}=\left(ae,bf,cg\right),a\geq 0,e\geq 0. \end{array}$$
(3) The product of any real number k and fuzzy number ⊗ is as follows:
  (28) $$\begin{array}{c} k⊗\tilde{A}=\left(\mathrm{ka},kb,kc\right),a\geq 0,k\geq 0. \end{array}$$
(4) The quotient of two fuzzy numbers ÷ is as follows:
  (29) $$\begin{array}{c} \tilde{A}÷\tilde{B}=\frac{a}{g},\frac{b}{f},\frac{c}{e},a\geq 0,\;e\geq 0 \end{array}$$

*(3) Linguistic Variables and Fuzzy Numbers*. Because language variables cannot be mathematically performed directly, the fuzzy number associated with each language variable describes the meaning of each generic language item. In the fuzzy set theory, language variables are converted to fuzzy numbers using language conversion scales. Determining the number of conversion scales is often intuitive: too few conversion scales will reduce the ability to differentiate, and too many conversion scales will make the system too complex and impractical. Miler (1965) states that the “seven plus or minus two” scale derives from the decision maker's absolute judgment-based goal that will produce the maximum amount of information, and seven transformation scales will be used in this article.

Because of the ambiguity of some indicators of judgment compliance, linguistic variables will be used in this paper to represent the important weights and alternative evaluation levels of a single criterion, as shown in Table 2 below.

**Figure 5:**
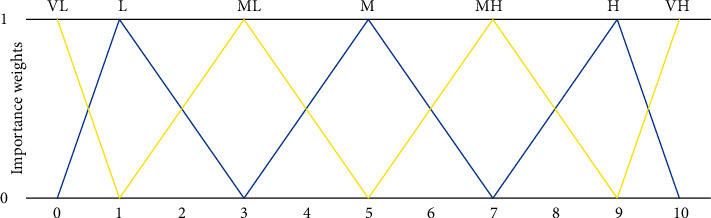
Membership function of important weight language variables.

**Figure 6:**
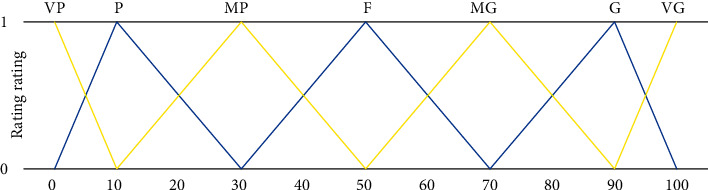
Membership function of evaluation grade language variables.

The membership functions of the language variables of important weight and evaluation level are shown in Figures 5 and 6.

*(4) Clarity Method of Fuzzy Number*. In this paper, the triangular fuzzy number represents the important weight and evaluation level in the evaluation index system. When applying the Shenfu group decision evaluation method, the fuzzy number must be converted into a clear real number to obtain the ranking of the selected schemes, i.e., clarity. The four most famous clarity methods are the center of gravity method, *α* maximum mean method, cut set method, and symbolic distance method. Each method has its own advantages and disadvantages. Here, the simplest and most widely used symbolic distance method is adopted. Using the symbolic distance method, the triangular fuzzy number $\tilde{A}$ = (a, B, *c*) is clearly expressed as d $\left(\tilde{A}\right)$.

(30) $$\begin{array}{c} d\left(\tilde{A}\right)=\frac{1}{4}\left(a+2b+c\right). \end{array}$$

### 4.4. Grey Neural Network Prediction Steps of Judgment Service Rate

1. Establish a multidimensional grey model gm1, 18 composed of 8 independent variables: case settlement rate, the completion rate of execution, the satisfaction rate of the plaintiff, the satisfaction rate of the defendant, litigation time, property preservation cycle, document delivery time, the disclosure rate of execution information, and 1 dependent variable: judgment service rate.
2. Normalize and accumulate the sample data of ABC court judgment service rate and its influencing factors from 2008 to 2020. The processed data is used as the training data and test data of the grey neural network.
3. Initialize the parameter a = B1 = B2 = B3 = B4 = B5 = B6 = B7 = B8 in the grey neural network as a random number between 0.32 and 0.55.
4. Initialize the learning rate U1 = U2 = U3 = U4 = U5 = U6 = U7 = *U*8 = 0.0015 in the grey neural network.
5. Initialize the weights and thresholds in the grey neural network.
6. Take 7 groups of data from 2008 to 2014 as training data and 6 groups of data from 2015 to 2020 as test data.
7. Set the number of cycle iterations in the grey neural network to 200, set the weight correction function and threshold correction function, set the stimulation function from the LA layer to the LB layer as a sigmoid function, and set the stimulation function between other layers as a purelin linear function.
8. The grey neural network uses seven groups of training data from 2008 to 2014 for self-learning and self-adaptation and constantly modifies the weights and thresholds until the minimum error target or the maximum learning times are reached to establish the grey neural network model of the judgment service rate of the ABC court.
9. Using the established grey neural network model of the judgment service rate of the ABC court, this paper forecasts the judgment service rate of the ABC court from 2015 to 2020 and compares the predicted value with six groups of test data from 2015 to 2020.

Matlab 2018b simulation software is used to program the multi-dimensional grey neural network model GM (1, 1) according to the grey neural network algorithm flow of ABC court judgment service rate, and the program is run. The running results show that the parameters of the grey neural network are as follows: w11 = 4.7765, w21 = −0.7523, w22 = 0.8363, w23 = 0.3224, w24 = 0.0109, w25 = 0.1506, w26 = 0.2937, w27 = 0.4706, w28 = 0.1545, w31 = w32 = w33 = w34 = w35 = w36 = w37 = w38 = w39 = 1.5908, a = 0.5262, *b*1 = 0.5449, *b*2 = 0.4097, *b*3 = 0.3278, *b*4 = 0.3645, *b*5 = 0.4022, *b*6 = 0.4487, *b*7 = 0.3656, LB_*b* = 1, LC_c 1 = −0.7523, LC_c 2 = 9.4950, LC_c 3 = 3.6337, LC_c 4 = 0.1235, LC_c 5 = 1.3719, LC_c 6 = 2.3498, LC_c 7 = 4.0003, LC_c 8 = 1.8001, LD_*d* = 38.5333, theta = 10.6348.

From the grey neural network model formula,

The grey neural network prediction model of the ABC court decision service rate is obtained.

## 5. Discussion

The compliance rate of the first instance judgment is an important evaluation index in the effect index of the information ball trial management system of the municipal high court. The evaluation of the compliance rate of the first instance judgment is of great help to the improvement of the trial management quality of the middle and grass-roots courts. Therefore, the prediction of the compliance rate of the judgment can provide reference for the trial of the court, which is the starting point of this study.

**Figure 7:**
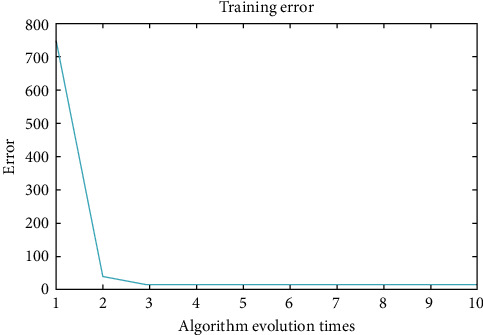
Algorithm evolution times.

Combined with relevant data and codes, the BP neural network is constructed for learning and training. The final training results are shown in Figure 7. The BP neural network reaches the minimum error value after running 10 times and stops iteration to obtain the optimal result.

**Figure 8:**
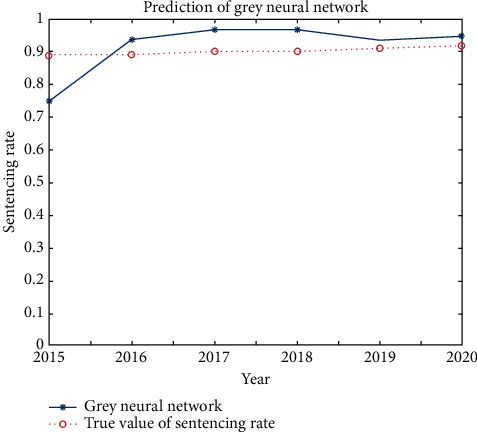
Comparison between the grey neural network prediction and actual sentencing rate.

By comparing the prediction model of the ABC court judgment service rate with the ABC court judgment service rate from 2015 to 2020, the comparison diagram of the prediction results of the ABC court judgment service rate is obtained as shown in Figure 8. It can be seen from Figure 8 that the fitting effect between the predicted value of the grey neural network and the actual judgment service rate is good.

Table 3 shows the comparison between the grey neural network and the ABC court judgment service rate from 2015 to 2020. It can be seen from Table 3 that the prediction results of the grey neural network are relatively accurate. The minimum relative error between the predicted value of the grey neural network and the actual judgment compliance rate is 2.38%, the maximum relative error is 13.9%, and the average relative error is 6.30%.

## 6. Conclusion

It is a common method to predict the future by calculating the evolution trend of historical data. However, because the judgment obedience rate is closely related to many factors and China is in an era of rapid development of social productivity, there is a certain gap between historical data and data in recent years. The prediction based on historical data has some limitations. Based on the combination of the grey network and BP neural network, this study alleviates this limitation to a certain extent, however, the changes that may be faced in the actual work are more special and complex. Therefore, how to predict the decision obedience rate more accurately in the future work is a proposition worthy of in-depth exploration.

Based on the grey network and BP neural network, the grey neural network model is established, and the judgment service rate of the ABC court is predicted based on 8 influencing factors. The simulation results show that the grey neural network model has a good simulation effect and small error. The prediction results show that the grey neural network prediction results have high accuracy and can obtain better prediction results. The main reason for obtaining high-precision prediction results is that the grey neural network combines the advantages of the BP neural network and grey system. The ABC court can optimize the allocation and reform of relevant resources according to the prediction of judgment service rate by the grey neural network to promote the development of the court and society.

## Figures and Tables

**Table 1:** Statistics of ABC courts from 2008 to 2020.

Year	Case closure rate (%)	Completion rate (%)	Plaintiff satisfaction rate (%)	Defendant satisfaction rate (%)	Litigation time/year	Property preservation period/day	Time/day of delivery of documents	Implementation of information disclosure rates (%)	Sentencing rate (%)
2008	57.7	68	67.3	65.5	1.2	720	13	65	86.4
2009	58.6	69.2	68.2	67.2	1.8	360	14	67	89.8
2010	60.1	70.3	69.4	68.8	1.5	720	10	71	88.7
2011	61.2	70.9	70.5	70.5	1.1	360	11	63	90.7
2012	61.5	71.2	71.8	70.6	1.4	360	12	68	87.8
2013	62.3	71.6	72.4	71.5	0.9	180	8	67	90.3
2014	62.8	73.3	72.6	72.5	1.3	720	10	80	86.9
2015	63	77.2	73.1	73.2	1.5	180	9	71	88.9
2016	63.8	80.4	73.6	74.8	1.2	180	7	73	89.0
2017	64.1	84.2	74.6	75.2	1.1	360	9	75	89.8
2018	65.3	85.4	76.2	77.4	1.5	720	6	75	90.2
2019	67.2	86.3	79.3	80.2	0.8	180	6	77	91.2
2020	76.7	87.7	84.6	84.4	1.1	720	4	80	91.7

**Table 2:** Linguistic variables and triangular fuzzy numbers for important weights and ratings.

Important weights	Rating		
Language variables	Triangular fuzzy number	Language variables	Triangular fuzzy number
Very low (VL)	(0, 0, 1)	Very poor (VP)	(0, 0, 10)
Low (L)	(0, 1, 3)	Poor (P)	(0, 10, 30)
Medium low (ML)	(1, 3, 5)	Medium poor (MP)	(10, 30, 50)
Medium (M)	(3, 5, 7)	Fair (F)	(30, 50, 70)
Medium high (MH)	(5, 7, 9)	Medium good (MG)	(50, 70, 90)
High (H)	(7, 9, 10)	Good (G)	(70, 90, 100)
Very High (VH)	(9, 10, 10)	Very good (VG)	(90, 100, 100)

**Table 3:** The difference between the actual compliance rate and the predicted compliance rate.

Year	Actual obedience to the verdict rate (%)	Predicted obedience to the verdict rate (%)	Difference (%)
2015	88.9	75.00	13.9
2016	89.0	94.03	5.03
2017	89.8	96.96	7.16
2018	90.2	96.42	6.22
2019	91.2	93.58	2.38
2020	91.7	94.80	3.10

## Data Availability

The data used to support the findings of this study are available from the corresponding author upon request.
